# Non-animal models in undergraduate education: opportunities, applications, and future directions

**DOI:** 10.1093/immhor/vlag032

**Published:** 2026-07-22

**Authors:** Sarah Teakel, Joanne Connolly, Thiru Vanniasinkam, Maurizio Costabile

**Affiliations:** School of Agricultural, Environmental and Veterinary Sciences, Charles Sturt University, NSW, Australia; School of Agricultural, Environmental and Veterinary Sciences, Charles Sturt University, NSW, Australia; School of Dentistry and Medical Sciences, Charles Sturt University, Sydney, NSW, Australia; School of Pharmacy and Biomedical Sciences, Adelaide University, Adelaide, South Australia, Adelaide, SA 5001, Australia; Higher Education Research Network, Adelaide University, Adelaide, South Australia, Australia

**Keywords:** CUREs, education, immunology, non-animal models, simulations, simulation based education

## Abstract

The use of animals in education and research has received increased scrutiny due to ethical and pedagogical considerations. Researchers have used non-animal models in immunology, medical, and veterinary disciplines for decades. Conversely, in education, there has been a reduced focus on teaching the theoretical and practical applications of these technologies. This has resulted in a gap between research and practice, limiting student exposure to the potential advantages of these technologies. In the last few decades, as non-animal technologies have become more sophisticated and widely available, the traditional reliance on animal models has slowly declined. With the emergence of new approach methodologies (NAMs), including organoids, organ-on-chip systems, bioprinting, computer simulations and other interactive digital tools, these offer authentic experiences while significantly reducing the need for animal use. Technologies such as organoids and computer simulations are now being increasingly introduced into undergraduate education. This “On The Horizons” narrative examines current non-animal model technologies, their benefits and limitations, and their implications for the evolving landscape of higher education. Importantly, the technologies covered in this paper have the potential to improve the student experience in programs that traditionally require the use of animals.

## Background

### Introduction

Undergraduate student research experiences enable the development of technical skills and facilitate the development of critical thinking, problem-solving skills, and bridging theory and practice. In the biological sciences, such as immunology, animal models are used to teach core concepts. However, the use of animals in education is increasingly questioned due to ethical, financial, and pedagogical considerations. Where there previously was a reliance on animals to teach, alternate strategies are now required to ensure that students continue to appreciate the relationship between core concepts and immunological settings while moving away from animal use. These approaches provide an alternate way to deliver authentic teaching experiences without the ethical and logistical complexities of animal use. Beyond teaching, limitations of animal models in research have driven the development of non-animal systems, including cell-based assays and computer simulations. However, barriers to accessibility have limited effective integration of these technologies into undergraduate teaching. In this article, we explore non-animal technologies currently being used in research, their potential application in undergraduate education, and the benefits and limitations of these approaches. The potential for non-animal systems to democratize access, enhance skill development, and promote ethically responsible science are explored in this paper. Finally, we focus on simulations as a viable alternative to non-animal models in teaching and research, noting that many non-animal technologies such as organoids and organs-on-chips may be unaffordable in some education settings.

Over recent decades, scientists have increasingly emphasized the importance of evaluating animal models used in research using the 3Rs, *Reduction, Refinement, and Replacement*, with the aim of minimizing animal use in research where possible and refining practices to reduce animal harm or distress. More recently, the principle of *Responsibility* has been introduced to promote humane care, respect and rehabilitation where reduction and replacement is not practical.^1^ However, there are limitations to non-animal technologies, which means it is unlikely that animal models will be entirely replaced by non-animal models in the near future. Since 1959, when Russell and Birch proposed a humane approach to research involving animal studies,^2^ the debate on improving and/or replacing animal models in research has continued. One reason for this is that scientists appreciate that while basic safety studies are useful in animal models, there can be significant issues when data obtained using animal models are translated or extrapolated across species. This lack of translatability and ethical concerns^3^ has led to the development of alternatives to animal models.

### Overview of traditional animal models

#### Types of animal models

Animal models have been central to biology education and research. Invertebrate models such as *Drosophila melanogaster* and *Caenorhabditis elegans* are extensively used in genetics, developmental biology, and neurobiology.^4–6^ Vertebrates, zebrafish (*Danio rerio*),^7^ and frogs (*Xenopus laevis)*^8^ are used in developmental and regenerative biology. Some of the most common animals used in immunological research are mice and rats, due to their well-characterized physiology and ability to readily manipulate their genotype.^9–10^ Larger mammals, including pigs and non-human primates, play important roles in translational research, although due to their size, housing requirements and specialized equipment required, their use is largely restricted to specialized laboratories.^11^

#### Applications in research and education

Animal models have been central to immunology, medical, and veterinary research for many years. Testing in animal models is the accepted gold standard, particularly in drug and vaccine development.^12^ In undergraduate education, animals are typically used to demonstrate anatomical features (eg spleen and lymph nodes), physiology (cardiovascular or respiratory models in rodents), or pharmacology (drug testing in zebrafish embryos).

#### Limitations in undergraduate contexts

The use of animal models can be justified in several undergraduate programs including animal and veterinary science, farm production, and agriculture, and in the introduction to laboratory research. However, the use of animals poses significant challenges, such as ethical approval processes, high maintenance costs, and student discomfort with dissection or live experimentation.^12–14^ Ethical considerations require regulatory oversight through animal ethics committees such as Institutional Animal Care and Use Committees (IACUC). Limited access to diverse species and variability in specimen quality can hinder equitable learning outcomes across institutions, and the cost of housing, breeding, and caring for animals can also be prohibitive for smaller institutions. Finally, while animal models provide biological realism, they may not align with pedagogical approaches, where repeatable access to animals in a low-risk setting may not be possible, especially for large class settings. These constraints reduce hands-on opportunities, pushing educators toward virtual or non-animal alternatives that maintain engagement while respecting welfare standards.

As concerns over using animals in research increases, educators have an important role to play in enhancing awareness of animal use in research to students. There is a critical need for undergraduate programs to include content that enhances students’ understanding of how skills they acquire in their program can be applied in their future career. For many STEM disciplines especially those that are biology-based, research undertaken in animal models continues to be an important part of the curriculum to provide a foundation for research at a graduate level. The importance of animal models for research is widely acknowledged.^15^ Despite some recent changes in animal testing requirements and regulations by the Food and Drug Administration (FDA) and the “2022 FDA Modernization Act 2.0”, it is still a requirement in many countries to conduct animal testing to demonstrate the safety and efficacy of novel drugs and vaccines prior to testing in human clinical trials.^16^

### Educational drivers

When introducing concepts related to animal research and the use of animals in research, it is imperative that relevant learning theory is considered in program design.

#### Teaching pedagogies in biological and veterinary sciences

Hands-on learning is central to both laboratory and animal use and is strongly supported by active learning frameworks. Active learning emphasises student engagement through doing, problem-solving, and reflection, rather than passive reception of information.^17^ These principles are particularly relevant in biological and veterinary sciences, where students must develop both practical skills and clinical reasoning in authentic contexts. Students are encouraged to engage with the content, apply concepts, and reflect on their experiences. These processes are fundamental to the development of scientific and clinical competence.

Active learning is most effective when students connect new information to prior knowledge, and when learning is situated in tasks that reflect real-world professional practice.^18^ In laboratory and animal-based teaching, this is achieved through direct engagement with procedures, equipment, and living systems. However, as the landscape of biological and veterinary education evolves, driven by both ethical considerations around animal use and advances in educational technology, a broader range of active learning strategies has emerged to complement and, in some cases, replace traditional approaches.

Virtual laboratories, case studies, simulations, and problem-based learning are all consistent with active learning principles, fostering critical thinking and meaningful engagement with course material.^19^ These approaches allow students to engage with complex, realistic scenarios in a safe and repeatable environment, supporting skill development without the constraints associated with live animal use. Simulations in particular offer opportunities for students to practice procedures, make decisions, and experience consequences in ways that reinforce learning and build confidence before real-world application.

In veterinary science education, technology-driven methods increasingly complement traditional practices, and evidence supports their effectiveness in improving student outcomes. The focus should remain on how technology can enhance student experiences by bridging theoretical knowledge with real-world application, ensuring that pedagogical innovation serves the broader goals of the program. Where possible, these tools are most effective when integrated into a blended learning environment, used alongside rather than in isolation from face-to-face teaching and practical experience.

### Emergence of non-animal models

**Figure 1:**
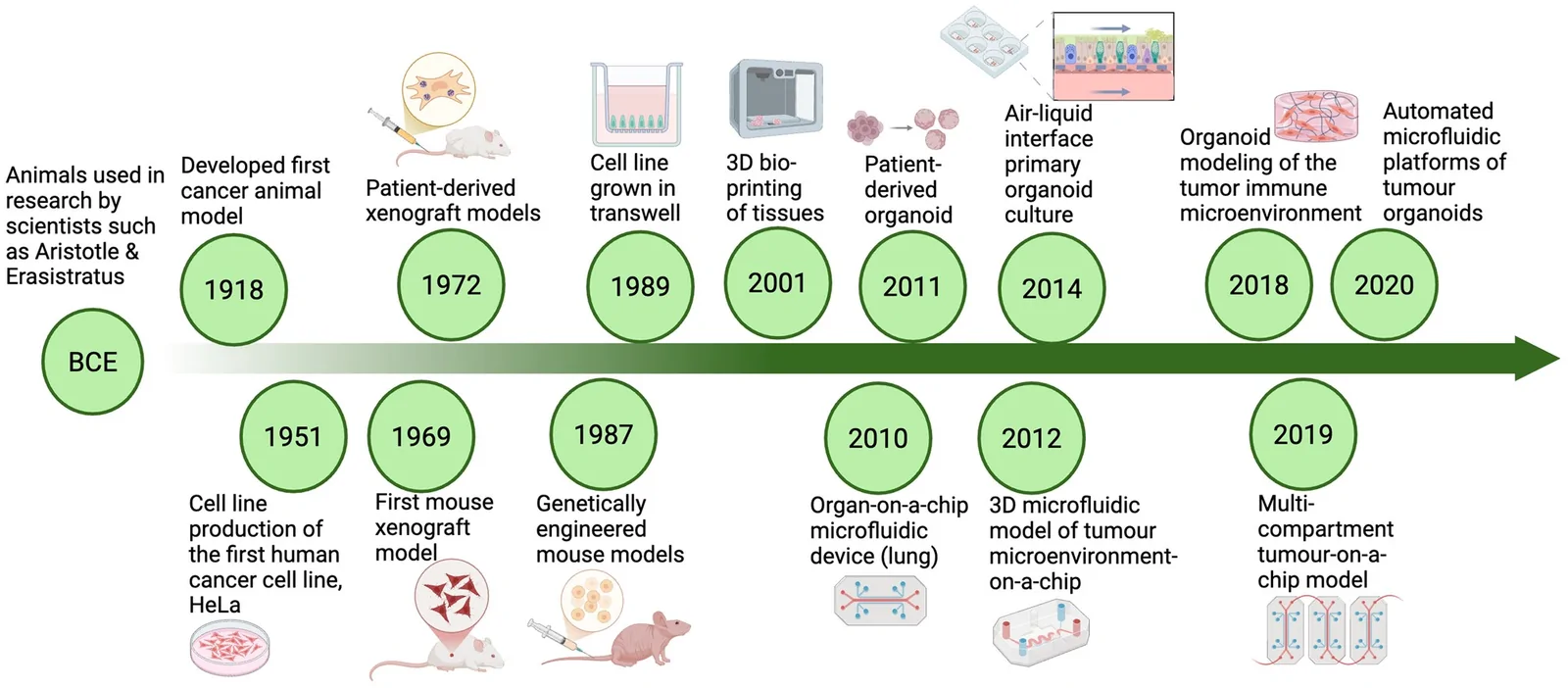
Evolution of experimental models from animal use to advanced cell-based technologies. Created in BioRender. Teakel, S. (2026) https://BioRender.com/nbzrjx4.

With the focus on reduction rather than replacing animal models, scientists have investigated cell culture, invertebrate models and other approaches for decades.^20^ More recently, with the advent of 3D cell culture, technologies such as organoids and organs-on-chips have been developed. From early reliance on animals to contemporary cell-based technologies, there has been a rapid progression of experimental models in biomedical research (Fig. 1). Key milestones include the development of immortalized cell lines (1951;^21^), xenograft models (1969–1972;^22^), and genetically engineered mouse models (1987;^23^), followed by innovations in cell culture systems,^24^ 3D bioprinting (2001;^25^), organ-on-a-chip platforms (2010;^26^) and organoid technologies.^27^ More recent advances automated microfluidic systems and multi-compartment tumor-on-chip models,^28–32^ with future directions pointing toward AI-driven simulations as scalable alternatives.^33^

#### Bioprinting

3D bioprinting has revolutionized laboratory-based research. However, despite increasing availability, it is not widely used due to technical, ethical, biological, and financial limitations. Bioprinting is a biomanufacturing technique that has potential for use in the design of a functioning organ^34^ with specific spatial architecture. In bioprinting, biomaterials such as cells are used to build tissues and organs. It should be noted that cells are sourced in an ethical manner from donors and their permission is granted before the cells are immortalized, and subsequently used in this approach. There are four major approaches; inkjet-based, laser-assisted, extrusion-based, and photo-curing bioprinting (Table 1).^35^ Bioprinting has the potential to improve any cell- and tissue-based technologies such as organoids to enable higher throughput.

**Table 1:** Features of bioprinting technologies.

Technology	Features
**Inkjet-based bioprinting**	*Deposits liquid bioink droplets using thermal or piezoelectric nozzles.* Capacity for multiple nozzles Requires liquid biomaterials
**Laser-assisted bioprinting**	*Uses laser pulses to position cells.* Does not cause mechanical damage to cells (high survival rates >95%) Can print multiple biomaterials including high-viscosity bioinks
**Extrusion-based bioprinting**	*Extrudes continuous filaments of high-viscosity biomaterials for printing.* Commonly used approach with a wide range of biocompatible materials However, high mechanical pressure and shear stress results in low cell viability
**Photo-curing bioprinting**	*Solidifies bioink layer-by-layer using light-induced cross-linking.* High efficiency and user friendly

#### Organoids and organ-on-a-chip

Organoids are 3D cell structures created in vitro from specific cell types or from stem cells, including induced pluripotent stem cells (iPSCs) or embryonic stem cells (ESCs) to generate specific cell types through targeted induction and differentiation protocols.^36^ 3D printing has also enabled scaffolding to form organoids with defined structures and functions. These structures mimic the architecture and function of a target organ or tissue and can be cultivated long-term in a laboratory setting, making them ideal for use in course-based undergraduate research experiences (CUREs), which may have previously required an animal model. Advances made in other fields including 3D printing, microfluidics, and genomics have also contributed to the development of organoids. Organoids are an alternative to animal models, offering researchers the ability to conduct studies involving differentiated cell types within an organ with dynamic cell–cell and cell–matrix interactions without the need for a live animal.^37^ In recent years organoids have been successfully used in various studies such as drug development, regenerative medicine, disease modeling, and precision medicine.^38–40^ Organ-on-a-chip technologies use small devices that mimic the functions of organs, however, they cannot entirely replicate the complex structural and cellular organization of organs. In contrast, organoids are 3D cell structures created *in vitro* from stem cells or include a mix of cell types.^41^ These structures can closely mimic the architecture and function of the target organ or tissue, maintain stable genetic traits, and can be cultivated long-term in a laboratory setting.^42–43^ Issues with developing vascularization of organoids and organs on chips that closely resemble the vasculature of a fully functioning organ within a host has been one of the most important factors that has hampered progress in the widespread use of these models in research. While many organoid and organ-on-chip models are often used successfully with microfluidic perfusions, in many cases this does not mimic the complex vasculature of an organ, and it is also often complex to set up microfluidic perfusions in experiments. More recently, significant progress has been made in advancing this technology, with researchers being able to develop pluripotent stem cell-based blood vessel organoids and a network of microvasculature around spheroidal organoids.^44^ These developments look promising and may help advance non-animal model technologies into the future.

#### Benefits and limitations of non-animal models in teaching

With recent advancements in organoid, organ-on-chip, and bioprinting technologies in research, there is the opportunity to extend their application to teaching. These platforms hold significant potential for modeling and predicting normal physiological processes and to enhance educational experiences. The effective teaching of non-animal models can be achieved within a laboratory environment, where students can engage directly with experimental systems. This context provides an opportunity to adopt a constructivist approach, designing activities that promote active learning and collaborative engagement. Ideally, such programs should enable students to develop hands-on skills while working with peers, fostering both technical competence and teamwork. Importantly, exposure to these approaches equips students with an understanding of the ethical use of non-animal models, a competency increasingly relevant in modern research environments. However, despite their promise, organoids and microfluidic chips remain costly, technically complex, and dependent on specialized facilities. Additional limitations of bioprinting are related to constraints of biomaterial, including resolution of printing and low cell viability.^45^

**Figure 2:**
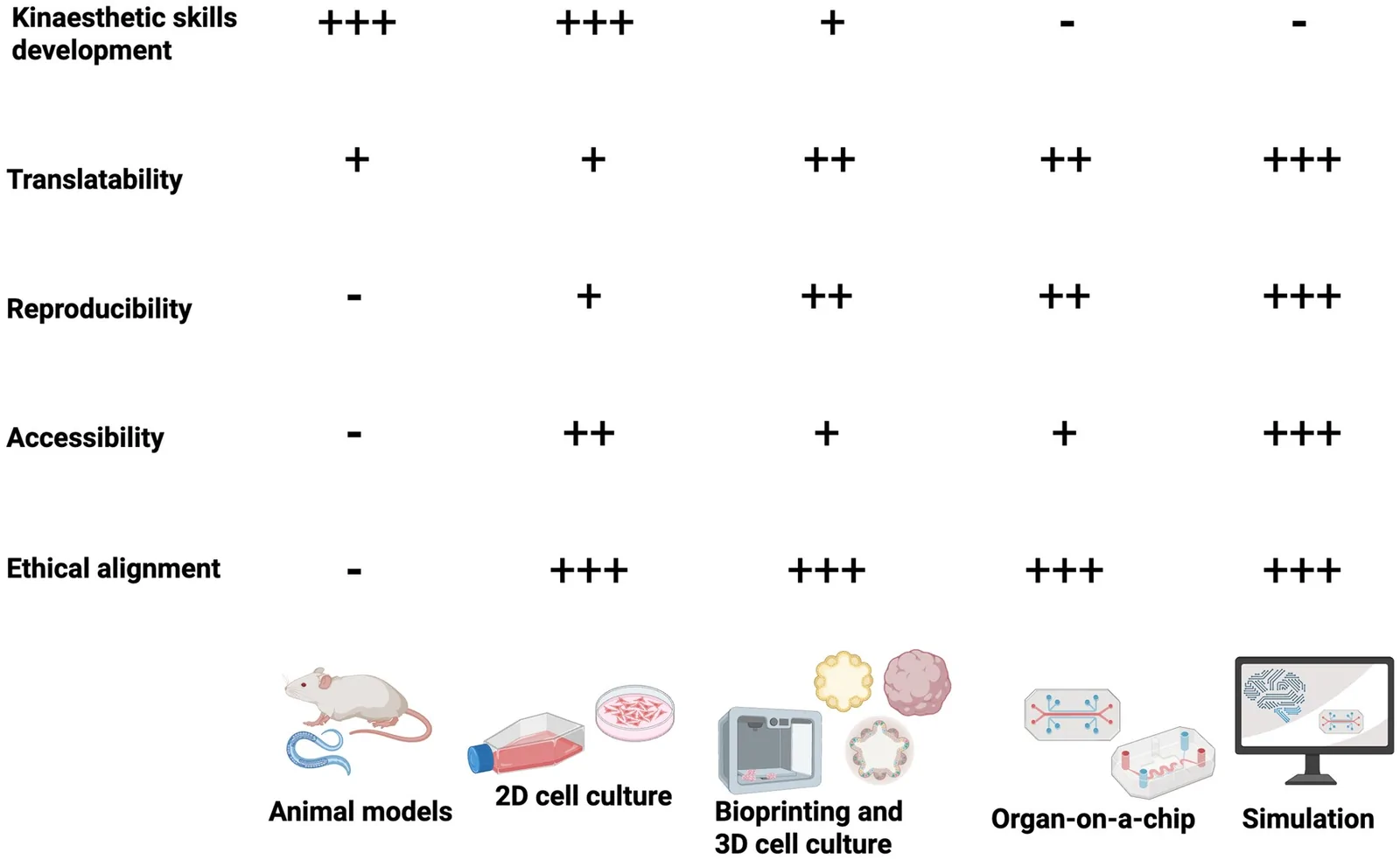
Comparing characteristics of traditional animal models to alternative non-animal models. Created in BioRender. Teakel, S. (2026) https://BioRender.com/nbzrjx4.

The advantages and disadvantages of animal models and non-animal models are compared in Fig. 2. Some requirements pose substantial barriers to their integration into undergraduate teaching. To aid the reader, we have defined educational translatability as how effectively non-animal models support applied learning outcomes in higher education. Reproducibility is related to the ability to consistently demonstrate expected laboratory or system outcomes to students. Accessibility relates to the extent to which non-animal models are readily available, affordable, and usable without specialised facilities or technologies. In the absence of standardised measures, it is evident that substantial disparities exist between institutions in the learning experiences available to students. Scalability is further restricted by the necessary skills and time required for preparation. Ethical alignment was informed by established international ethical frameworks. However, ethical alignment is context-dependent and must align with relevant national and institutional standards without broad generalisation. Here, we also introduce some advantages of simulations as alternatives to animal use.

### Virtual and simulation-based approaches

One approach to remove the use of animal models yet still deliver undergraduate training is simulation-based learning (SBL). Educators must balance authenticity with accessibility and practicality, often employing simplified representations or simulations to convey complex concepts effectively. Where laboratory access is restricted, simulations based on organoid technologies offer viable alternatives. These strategies support long-term retention, facilitate the application of theoretical knowledge to practice, and provide a strong foundation for subsequent programs involving animal use. Additionally, simulations allow real-time visualisation of molecular processes that would otherwise require advanced microscopy or specialized equipment, thereby enhancing comprehension of physiological mechanisms. In previous studies, we have successfully used simulations to teach a range of content and laboratory concepts in immunology,^46–48^ microbiology^49^ and genetics^50^ disciplines. These approaches have led to significant educational improvements in student learning in all disciplines tested.

#### Proposed benefits of simulation-based models

Simulations have been effectively used to overcome cost and physical limitations associated with equipment, safety, and use of animals in the training of undergraduate students. SBL has emerged as a highly effective strategy in health, medical, and STEM education. It provides safe, scalable, and immersive environments for learners to develop technical skills and critical thinking. It allows students to engage with complex technologies and procedures in a risk-free setting, enabling repeated practice with immediate feedback.^51–52^

Virtual laboratories and simulation software allow students to conduct experiments in silico, often with immersive visualizations. Platforms, such as Labster (www.labster.com/) or PhET (https://phet.colorado.edu/), simulate experimental workflows ranging from molecular biology techniques to physiology experiments. Augmented reality dissections and virtual microscopy provide interactive experiences that mimic traditional laboratory work, often at lower cost and without ethical complications.

SBL is a well-accepted pedagogical approach that complements traditional methods by bridging theory with practice and enhancing learner engagement and outcomes. Studies have consistently demonstrated that SBL enhances knowledge acquisition, procedural competency, and learner confidence across diverse educational domains. In undergraduate medical education, SBL has been successfully implemented in anatomy, where interactive 3D virtual simulations improve spatial understanding of anatomical structures. While in basic sciences, such as biochemistry, genetics, hematology, histology, immunology, and microbiology, simulations and immersive software enhance student learning and engagement with the content.^46^

#### Evaluating the educational effectiveness of non-animal models

The effectiveness of non-animal models in undergraduate education must be evaluated through the lens of achieving equivalent or improved learning outcomes compared to traditional animal-based approaches. Assessment strategies, therefore, play an important role in determining whether technologies such as simulations, virtual laboratories, and organoid-based teaching activities provide pedagogically valid replacements. Pre- and post-intervention designs are particularly well suited to this investigation, enabling direct measurement of knowledge gains associated with specific non-animal interventions.

For example, simulation-based learning environments enable formative assessments to be embedded at interactive decision points, allowing for the evaluation of conceptual understanding and procedural competence. Virtual laboratory platforms can similarly capture student progression, error rates, and application of knowledge, providing detailed insight into learning processes that are often difficult to measure in traditional animal-based practical classes.

Assessment modalities such as laboratory reports, simulation-embedded quizzes, and structured examinations can be used to compare learning outcomes between animal-based and non-animal approaches. The pre- and post-assessment approach allows for the collection of additional quantitative data, beyond traditional examination scores, enabling measurement of changes in both knowledge and student perceptions. In addition, the inclusion of free-text responses provides valuable qualitative insight into student experiences, including aspects that were effective, areas for improvement, and suggested enhancements that may not be apparent through quantitative measures alone. The provision of structured assessment opportunities also supports students in monitoring their own learning progression.

A key advantage of simulation-based models is the integration of embedded assessment, enabling both formative and summative evaluation within the learning environment. When implemented within a blended learning framework, simulations are particularly effective as preparatory activities prior to face-to-face instruction. Evidence indicates that such pre-learning approaches can enhance student understanding and reduce cognitive load during subsequent in-person sessions.^53–54^

Collectively, these approaches provide a framework for rigorously evaluating whether non-animal models can achieve comparable or enhanced educational outcomes relative to traditional animal-based teaching.

#### Future of simulations in higher education

While promising, simulations currently complement rather than replace experimental research. They model conditions based on existing data but lack full biological complexity. Their predictive accuracy depends on robust datasets and algorithms, areas requiring further research and validation. They hold potential for hypothesis testing and preclinical screening. Artificial Intelligence (AI) has revolutionized personalized medicine and drug discovery through virtual screening with predictive software for protein structure and interactions.^55–57^ Overall, this has reduced the time and cost associated with traditional medical research. The use of simulations in training is increasing and is transforming medical education.^58^ Simulation-based learning has the potential to become the primary mode of training or replace certain wet lab learning entirely, especially as immersive, adaptive, and gamified platforms advance. The integration of AI within simulation platforms has the potential to enable researchers, educators, and students to visualize complex biological processes through tangible, interpretable outputs that can be applied in experimental design and education settings.

## Conclusion

The growing ethical, financial, and pedagogical challenges associated with the use of animals as models in education and research underscore the need for viable alternatives such as organoids, organ-on-chip systems, bioprinting, and simulation-based learning. Advances in non-animal technologies offer promising pathways to reduce reliance on animal models. These approaches address welfare concerns and increase accessibility. Complete replacement of animals use in high education remains unlikely. However, as non-animal technologies improve and become validated, accreditation bodies and ethics committees may increasingly encourage or mandate reductions in animal use, reshaping the expectations of future educators and researchers. Additionally, fostering ethical awareness ensures that future educators and researchers recognize the imperative to continue developing, adopting, and advocating for alternatives to animal use across teaching and research. Ultimately, the strategic adoption of these technologies represents a forward-looking approach that aligns with a One Health approach in STEM education and global trends toward responsible, sustainable development goals, preparing graduates for an evolving research landscape.
